# Presumed Retinal Metastasis from Lung Adenocarcinoma: A Case Report and Literature Review

**DOI:** 10.1155/2021/6615284

**Published:** 2021-06-10

**Authors:** Gökçen Özcan, Ahmet Kaan Gündüz, Ibadulla Mirzayev, Ayhan Sağlik

**Affiliations:** ^1^Ankara University Faculty of Medicine, Ophthalmology Department, Ankara, Turkey; ^2^Harran University Faculty of Medicine, Ophthalmology Department, Şanlıurfa, Turkey

## Abstract

A 63-year-old Caucasian man with metastatic lung adenocarcinoma undergoing chemotherapy and external radiotherapy was referred for routine eye examination. Although he was asymptomatic, ocular examination revealed a relatively well-circumscribed whitish retinal lesion measuring 0.5 × 0.5 × 0.5 mm located along the inferotemporal vascular arcade in the right eye. Optical coherence tomography (OCT) showed a hyperreflective dome-shaped lesion occupying the inner retinal layers with few hyperreflective dots overlying the retina in the posterior vitreous consistent with tumor cells. Fluorescein angiography revealed early hyperfluorescence and late staining without leakage at the lesion site. A diagnosis of presumed retinal metastasis from lung adenocarcinoma was made. At 2 months follow-up after completion of chemotherapy, the retinal lesion was found to have regressed completely leaving minor irregularities in the inner retinal layers on OCT. To date, there have been only 41 cases of carcinoma metastasis to the retina reported in the literature including the current case. Despite its rarity, retinal metastasis should be considered in the differential diagnosis of a white-yellow retinal mass with/without overlying vitreous cells especially in patients with a history of systemic cancer.

## 1. Introduction

Metastatic tumors of the uveal tract are the most common intraocular malignancies, whereas retinal metastases are rare compared to uveal metastases [1]. The choroid is the most common site of uveal metastasis. The choroid receives 85% of the ocular blood flow compared to the retina which receives only 5%, thereby explaining the rarity of retinal metastasis [2]. Carcinomas metastasize to the choroid more often compared to sarcomas and melanomas. However, melanomas tend to metastasize to the retina more frequently than carcinomas [3]. Tumor cells are periodically shed to the circulation from the primary site. Expression of specific adhesion molecules on extravasated metastatic cells allows the proliferation of these cells on the metastatic host sites [4]. Several previously published papers reviewed vitreous and retinal metastasis from cutaneous melanomas [5, 6]. We hereby present a case of retinal metastasis from lung carcinoma and review the literature on carcinoma metastasis to the retina.

## 2. Case Report

**Figure 1:**
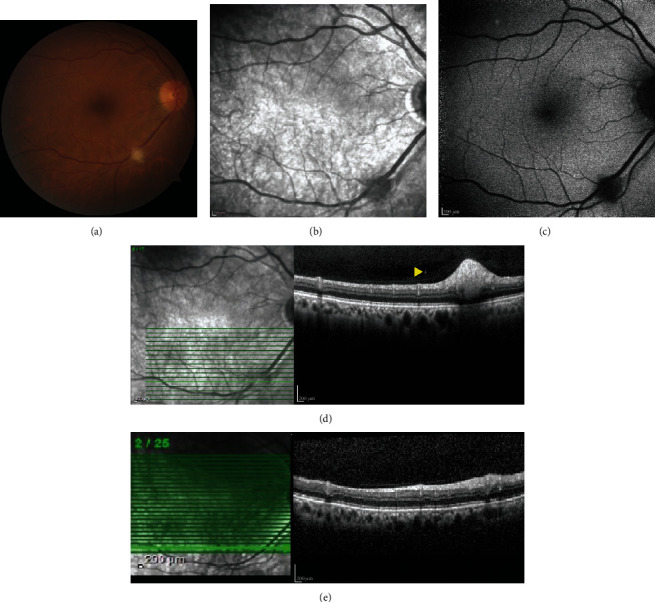
(a) Whitish perivascular retinal lesion measuring approximately 0.5 x 0.5 x 0.5 mm located along the inferotemporal arcade in the right eye. (b) The lesion is hyporeflective on infrared imaging. (c) The lesion shows hypoautofluorescence on fundus autofluorescence imaging. (d) Spectral domain optical coherence tomography (SD-OCT) imaging shows the hyperreflective dome-shaped lesion replacing inner retinal structures, causing disorganization and compression of the outer retina. Multiple hyperreflective dots are observed overlying the retina consistent with tumor cells in the posterior vitreous (arrowhead). Ellipsoid zone cannot be observed clearly because of partial posterior shadowing by the tumor, but the retinal pigment epithelium is intact. (e) At 2 months follow-up examination after completion of chemotherapy, the retinal lesion regressed completely leaving only focal irregularities in the inner retinal layers on SD-OCT.

A 63-year-old Caucasian man diagnosed with metastatic lung adenocarcinoma 5 months ago and receiving systemic chemotherapy and external radiotherapy to the right hip underwent routine eye examination. He had no history of known ocular disease and intervention and was asymptomatic. Best corrected visual acuities were 20/32 in the right eye and 20/20 in the left eye. Intraocular pressures were 15 mmHg and 17 mmHg in the right and left eyes, respectively. Anterior segment examination showed both eyes had moderate nuclear sclerosis. Fundus examination of the right eye revealed a relatively well-circumscribed whitish retinal lesion measuring 0.5 × 0.5 × 0.5 mm located along the inferotemporal vascular arcade. There was no apparent vitreous and choroidal involvement. The left eye had normal funduscopic findings with blunted foveal reflex. Spectral domain optical coherence tomography (SD-OCT) of the right eye showed a hyperreflective dome-shaped lesion occupying the inner retinal layers with few hyperreflective dots overlying the retina consistent with tumor cells in the posterior vitreous. The outer retina was compressed by tumor and partial choroidal shadowing was observed without clinical or imaging evidence for choroidal involvement. The lesion was hypofluorescent on fundus autofluorescence (FAF) imaging. Fluorescein angiography (FA) revealed early hyperfluorescence of the lesion, late staining, and no leakage. The tumor was flat and nonmeasurable on B-mode ultrasonography. Brain magnetic resonance imaging revealed normal findings. Based on the clinical and imaging features, a diagnosis of presumed lung carcinoma metastasis to the retina was made. At a follow-up examination 2 months later after completion of systemic chemotherapy, the visual acuity in the right eye was 20/60 due to the worsening of the cataract. Fundus examination revealed that the retinal lesion in the right eye had regressed completely. There were only focal irregularities in the inner retinal layers on SD-OCT (Figure 1). The patient passed away 6 months after the diagnosis of retinal metastasis.

## 3. Discussion

Cutaneous melanoma is the most common metastatic retinal tumor. Tang et al. reviewed 42 cases of tumor metastasis to the retina published in the English literature from 1935 to 2017 and noted that the most common primary sites for metastatic tumors to the retina were cutaneous melanoma followed by lung carcinoma and gastrointestinal carcinoma [7]. Ramaesh et al. reported 39 melanoma metastases to the vitreous and retina in his literature review in 1998 [6]. Our search of the literature showed that there were at least 60 cases of melanoma metastasis to the vitreous and retina reported so far and the total number of reported carcinoma metastasis to the retina was 40. Of the 41 cases with carcinoma metastasis to the retina including our case, 15 (36.6%) cases had lung carcinoma, 13 (31.7%) cases had gastrointestinal carcinoma including 1 case of pancreatic carcinoma and 1 case of hepatocholangiocarcinoma, 6 (14.6%) had breast carcinoma, 3 (7.3%) had adenocarcinoma with unknown primary site, 2 (4.9%) had genitourinary tract carcinoma, 1 (2.4%) had uterine carcinoma, and 1 (2.4%) had nasopharyngeal carcinoma. The mean age of all reported cases was 56.1 (range 15-75, SD ± 11.9) years. Twenty-five out of 41 (61.0%) patients were males, and the remaining 16 (39.0%) were females.

Retinal metastases are usually unilateral and solitary; however, bilateral and multifocal involvement have also been reported [8–10]. At the time of diagnosis of retinal metastasis, the majority of patients have metastasis at other sites as well (Table 1). Alternatively, ocular involvement may be the first finding of metastatic disease, and these patients must be carefully evaluated for the presence of systemic metastases at other sites.

Decreased vision, floaters, and ocular pain are the common presenting features in carcinoma metastasis to the retina. However, retinal metastasis can be asymptomatic as in our case. This underlines the necessity for patients with metastatic systemic cancer to have periodic eye examinations. Carcinoma metastasis to the retina most commonly presents as a focal, yellow-white, intraretinal mass with ill-defined borders. However, the tumor borders can sometimes be relatively well-circumscribed, as in our case. Overlying vitreous cells can be observed mimicking uveitis. Vitreous involvement may be seen as white-yellow cellular clumps or strands, sometimes hindering the view of the retina. Sometimes anterior chamber cells and a pseudohypopyon formed by tumor cells can also be observed [9, 10]. Glaucoma secondary to the obstruction of aqueous outflow by tumor cells in the trabecular meshwork may be observed.

Metastatic retinal melanoma usually presents with a brown lesion with ill-defined borders, but amelanotic melanoma metastasis with yellow color has also been described. Vitreous metastasis from cutaneous melanoma is characterized by pigmented cellular clumps. On the other hand, retinal carcinoma metastasis is characterized by ill-defined whitish retinal infiltration with overlying nonpigmented cellular aggregates [11].

In our literature review of metastatic retinal cancer (Table 1), vitreous involvement in addition to retinal metastasis was found in 17 (41.5%) cases. Optic disc and choroidal involvement together with retinal metastasis were reported in 5 (12.2%) cases [8, 10, 12–15]. Retinal involvement usually starts from the retinal nerve fiber and ganglion cell layers. The superficial vascular plexus located in the ganglion cell layer and supplied by the central retinal artery is the major source of metastatic tumors to the retina. As retinal metastasis grows, it replaces deeper retinal tissue. After full-thickness retinal involvement occurs, tumor cells may break through the retina pigment epithelium/Bruchs membrane and invade the choroid [10, 16]. Tumor cells may also penetrate the inner limiting membrane and spread into the vitreous cavity. Isolated carcinoma metastases to the vitreous have also been reported; however, histopathologic findings of enucleated eyes with isolated carcinoma metastases to the vitreous showed microscopic retinal involvement [5, 17].

Retinal metastases tend to be angiocentric and grow around the vessels as they are bloodborne [14, 18]. Histopathologic examination shows tumor emboli in the lumens of retinal vessels in the ganglion cell layer around retinal infiltrations [5, 14, 17, 19]. Retinal vascular occlusion has been reported leading to retinal nonperfusion and neovascularization [10, 20]. Complete disappearance of retinal metastatic lesion after chemotherapy as in our case is rare and has been reported only once before in a 15-year-old boy with nasopharyngeal carcinoma [21].

Diagnostic methods used in retinal metastatic tumors include OCT, OCT angiography, ultrasonography, FAF, FA, fine needle aspiration biopsy, pars plana vitrectomy, and chorioretinal biopsy. The differential diagnosis of carcinoma metastasis to the retina includes cotton wool spots, retinal astrocytic hamartoma, inflammatory retinitis, and primary vitreoretinal lymphoma. The former 2 lesions originate from the retinal nerve fiber layer, whereas carcinoma metastasis to the retina demonstrates multilayer involvement of the retina. Nonpigmented vitreous cells also pose a diagnostic challenge as they can resemble vitreoretinal lymphoma, amyloidosis, candida endophthalmitis, or intermediate uveitis. Cellular aggregates in the vitreous appearing as spherules or strands should always alert the clinicians to the possibility of metastatic cells.

Treatment options for carcinoma metastasis to the retina include observation, systemic chemotherapy, intravitreal chemotherapy, plaque radiotherapy, external radiotherapy, photodynamic therapy, surgical excision, and enucleation for a blind, painful eye [22–24]. The survival rate for patients with carcinoma metastasis to the retina is poor, and the mean survival for all reported cases with retinal metastasis was only 5.7 (range 1-23, SD ± 5.2) months (Table 1).

In summary, carcinoma metastasis to the retina is rare with only 41 cases being reported so far including the current case. The lung is the most common primary site for carcinoma metastasis to the retina followed by the gastrointestinal system. Retinal metastases are angiocentric, originate from the superficial vascular plexus, and are usually initially located in the inner retina. Vitreous involvement may also be seen. Patients generally have widespread metastasis at the time of diagnosis, and the life expectancy is poor. Treatment options are varied and should be tailored to the patients' needs.

## 4. Method of Literature Search

The literature search consisted of a systematic review of the literature using PubMed database till January 2021 using the following key words: “retinal metastasis” or “carcinoma metastasis to the retina” or “metastatic carcinoma to the retina”. The reference lists of the index publications identified through PubMed were also evaluated and relevant papers were retrieved. Chapters on metastatic tumors to the retina in major ophthalmology textbooks were reviewed. Articles written in non-English languages were also included. A total of 40 cases with carcinoma metastasis to the retina were found and reviewed for this study.

## Figures and Tables

**Table 1:** Review of cases with metastatic retinal cancer reported in the literature.

	Author/year	Sex	Age	Primary tumor	Location	Primary tumor known at eye diagnosis	The onset of eye findings (mo)	Diagnosis	Other known systemic metastasis	Ocular treatment	Follow-up	Clinical signs/pathologic findings
1	Arisawa, 1914 [12] (cited by Kennedy et al., 1958 [20])	M	30	Pancreatic carcinoma	Diffuse	Yes	N/A	Autopsy?	N/A	N/A	N/A	Vitreous seeds, optic disc involvement
2	Smoleroff and Agatston, 1934 [18] (cited by Kennedy et al., 1958 [20])	M	55	Esophageal adenocarcinoma	Inferotemporal quadrant	Yes	N/A	Autopsy	Liver, lung, adrenal gland, spine	N/A	1 mo, deceased	White irregular mass arising from nerve fiber layer, retinal and subretinal but no choroidal involvement
3	Kennedy et al., 1958 [20]	M	51	Rectosigmoid adenocarcinoma	Macula	No	0	Enucleation	Brain	Enucleation	9 mo, deceased	Grayish white nodular lesion
4	Duke and Walsh, 1959 [16] (cited by Tang et al., 2019 [7])	F	60	Uterine adenocarcinoma	Macula	Yes	N/A	Enucleation	Brain	Enucleation	6 mo, deceased	Vitreous seeds, elevated white mass
5	Koenig et al., 1960 [10]	M	56	Undifferentiated bronchogenic carcinoma	Temporal retina	No	0	Enucleation	N/A	Enucleation	13 mo, deceased	Granulomatous uveitis, vitreous floaters, white mass with neovascularization, retinal and subretinal exudation, choroidal seeding
6	Flindall and Fleming, 1967 [25]	M	68	Unknown primary	Anterior to equator	No	N/A	Enucleation	N/A	Enucleation	24 mo, alive	Vitreous seeds, veil-like retinal exudates inferiorly, optic disc involvement
7	Levy and de Venecia, 1970 [14]	M	49	Oat cell carcinoma of lung	Posterior pole	Yes	10	Autopsy	Widespread	Observation	10 mo, deceased	Gross ophthalmic examination was normal; histopathology revealed anaplastic cells filling the lumens of retinal vessels, nerve fiber layer, and isolated choroid involvement
8	Klein et al., 1977 [8]	M	52	Squamous cell carcinoma of lung	Temporal macula OD, ERD OS	Yes	N/A	Autopsy	Widespread	Observation	2 mo, deceased	White retinal infiltrate, retinal choroidal, and optic disc involvement
9	Young et al., 1979 [26]	M	63	Lung adenocarcinoma	Macula	Yes	4	VAB and autopsy	Brain, bone	External radiotherapy	7 mo, deceased	Perivascular white infiltrates around optic disc, white macular mass
10	Piro et al., 1982 [17]	F	56	Breast carcinoma	Vitreous opacities OU	No	0	PPV and autopsy OD, autopsy OS	No	Observation	12 mo, deceased	Uveitis, vitreous seeds, no retinal infiltration; histopathology showed solitary tumor cells in retina
11	Eagle, 1988 [27] (cited by Srivastava and Bergstrom, 2013 [28])	F	53	Unknown primary	Macula	No	0	VAB, retinal and choroidal biopsy	Brain, lung	N/A	5 mo, deceased	Vitreous balls, white macular lesions with satellites, and vascular sheathing
12	Takagi et al., 1989 [19]	M	45	Adenocarcinoma of lung	Inferotemporal quadrant	Yes	24	Enucleation	Adrenal gland, rectum	Enucleation	3 mo, deceased	Vitreous seeds, white exudates along the retinal veins, optic disc involvement, tumor cell emboli in central retinal vein
13	Leys et al., 1990 [29]	M	49	Oat cell carcinoma of lung	Temporal macula	Yes	0	Autopsy	Brain, kidney, liver	Observation	1 mo, deceased	Vitreous seeds, ERD, partially obscured retinal arteriole
14	Striebel-Gerecke et al., 1992 [24] (cited by Srivastava and Bergstrom, 2013 [28])	F	47	Oat cell carcinoma of lung	Inferonasal quadrant	Yes	N/A	VAB, autopsy	Brain, liver, adrenal gland	N/A	2.5 mo, deceased	White cone shape mass in vitreous, plaque like retinal infiltration
15	Tachinami et al., 1992 [30] (cited by Srivastava and Bergstrom, 2013 [28])	M	61	Rectal adenocarcinoma	Superior macula	Yes	N/A	Autopsy	Brain, lymph nodes	N/A	5 mo, deceased	White retinal mass, ERD
16	Spraul et al., 1995 [31]	F	74	Muir-Torre syndrome/adenocarcinoma of lung or breast	Superotemporal quadrant	Yes	N/A	Enucleation	Lung	Enucleation	10 mo, alive	Yellow-white tumor, shallow ERD, adenocarcinoma cells involving the retina
17	Cangiarella et al., 1996 [32]	F	51	Esophageal adenocarcinoma	Unknown	No	0	VAB	Lymph nodes	N/A	4 mo, alive	Vitreous seeds, white retinal infiltrate, ERD
18	Hutchison et al., 2001 [33] (cited by Srivastava and Bergstrom, 2013 [28])	F	63	Colon adenocarcinoma	Superotemporal macula	Yes	N/A	N/A	Lung	N/A	3 mo, alive	Pale elevated vascular lesion superotemporal to macula, ERD
19	Gupta et al., 2002 [13]	M	46	Oat cell carcinoma of lung	Juxtapapillary	No	0	Clinical	Skin, lymph nodes	Chemotherapy only	1 mo, deceased	Pale elevated juxtapapillary lesion, necrosis, optic disc, retinal and choroidal involvement
20	Truong et al., 2002 [34]	F	59	Breast carcinoma	Temporal to fovea	Yes	36	Clinical	Brain, lung, liver	External radiotherapy	N/A	White retinal and subretinal mass, ERD
21	Saornil et al., 2004 [35]	M	70	Gastric adenocarcinoma	Juxtapapillary	Yes	192	Enucleation	Mediastinal lymph node	Enucleation	23 mo, deceased	White retinal tumor, iris neovascularization (neovascular glaucoma), ERD
22	Apte et al., 2005 [23]	M	39	Cecal adenocarcinoma	Inferior macula	Yes	3	PPV, excisional biopsy	Liver, lung	External radiotherapy	3 mo, alive	Vitreous seeds, ERD
23	Rossi et al., 2005 [36]	M	41	Non-small-cell lung carcinoma	Superotemporal macula OU	Yes	6	Clinical	Bone, brain	Chemotherapy only	3 mo, deceased	White elevated retinal mass, ERD
24	Rundle and Rennie, 2006 [22]	F	55	Breast carcinoma	Temporal to fovea	Yes	108	Clinical	Lung	Photodynamic therapy	2 mo, alive	White retinal mass, SRF
25	Sirimaharaj et al., 2006 [37]	F	60	Breast carcinoma	Nasal midperipheral retina	Yes	36	PPV	Spine, lung, brain	External radiotherapy	8 mo, deceased	Vitreous cell clumps, white precipitates vascular sheathing
26	Alegret et al., 2009 [21]	M	15	Nasopharyngeal carcinoma	Inferotemporal arcade	Yes	12	Clinical	Lung	Chemotherapy only	N/A	White nodular lesion
27	Kim et al., 2010 [38]	F	64	Gastric adenocarcinoma	Macula OU, vitreous OD	Yes	24	PPV	Lung, liver, peritoneal	External radiotherapy	1 mo, alive	Vitreous seeds, white nodular lesion
28	Coassin et al., 2011 [39]	F	54	Small cell lung carcinoma	Temporal macula	N/a	N/A	PPV, retinal biopsy	Brain	N/A	7 mo, alive	Vitreous seeds, white retinal infiltrates
29	Payne et al., 2012 [40]	M	62	Small cell lung carcinoma	Temporal macula	N/a	N/A	VAB, retinal biopsy, PPV	N/A	External radiotherapy	10 mo, alive	Vitreous haze, retinal whitening
30	Shields et al., 2014 [3]	F	75	Breast carcinoma	Inferotemporal quadrant	Yes	103	N/A	N/A	Observation	N/A	Yellow retinal mass, irregular contour, intrinsic hemorrhage, SRF
31	Shields et al., 2014 [3]	M	56	Esophageal adenocarcinoma	N/A	Yes	26	N/A	N/A	Observation	1 mo, deceased	Retinal whitening, SRF
32	Shields et al., 2014 [3]	F	58	Breast carcinoma	N/A	Yes	214	N/A	N/A	Observation	1 mo, deceased	Retinal whitening, SRF
33	Shields et al., 2014 [3]	F	64	Lung carcinoma	Temporal macula	Yes	20	Fine needle biopsy	N/A	Plaque radiotherapy	4 mo, deceased	White retinal mass, vitreous seeds, intrinsic vascularity, vitreous hemorrhage
34	Taubenslag et al., 2015 [20]	M	75	Non-small-cell lung carcinoma	Superotemporal quadrant	No	0	PPV, retinal biopsy	No	External radiotherapy	18 mo, alive	Branch retinal vein occlusion, SRF, vitreous cells, presumed optic disc involvement
35	Praidou et al., 2017 [41]	M	70	Hepatocholangio carcinoma	Inferotemporal arcade	Yes	11	PPV, retinal biopsy	Liver	Resection and chemotherapy	12 mo, alive	Vitreous seeds, white retinal lesion, SRF
36	Essadi et al., 2017 [42]	M	62	Clear cell renal carcinoma	N/A	Yes	28	Clinical	Lung	Chemotherapy only	4 mo, alive	Large retinal mass, retinal hemorrhage
37	Whalen et al., 2018 [9]	M	55	Bladder cancer	Inferonasal quadrant	Yes	48	PPV	No	External radiotherapy, enucleation	23 mo, alive	Uveitis, pseudohypopyon, SRF, and satellite lesions
38	Mano et al., 2018 [43]	M	69	Esophageal adenocarcinoma	Macula and peripheral retina	Yes	N/A	PPV	Widespread	Antiviral treatment	3 mo, deceased	Vitreous seeds, white infiltrates simulating acute retinal necrosis
39	Ramtohul et al., 2019 [44]	M	51	Colon adenocarcinoma	Superior macula	Yes	N/A	N/A	N/A	N/A	6 mo N/A	Large vascularized retinal mass, retinal hemorrhage (initially), choroidal involvement (at 6 mo follow-up)
40	Jorge et al., 2020 [15]	F	63	Lung carcinoma	Juxtapapillary	No	0	Clinical	Liver	Chemotherapy only	8 mo alive	Yellow retinal lesion

M: male; F: female; N/A: not available; SRF: subretinal fluid; ERD: exudative retinal detachment; VAB: vitreous aspiration biopsy; PPV: pars plana vitrectomy; OD: right eye; OS: left eye; OU: both eyes; mo: month.
